# Research Progress on the Pre-Treatment of Chicken Feathers for Biogas Production

**DOI:** 10.3390/bioengineering13090962

**Published:** 2026-08-23

**Authors:** Isa Beatriz Conceição Oliveira-Alves, Hortência E. P. Santana, Ingrid Vieira Fernandes, Meirielly Jesus, Joana Santos, Fernando Mata, Samia Tássia Andrade Maciel, Denise Santos Ruzene, Daniel Pereira Silva

**Affiliations:** 1 RENORBIO—Northeastern Biotechnology Network, Federal University of Sergipe, São Cristóvão 49107-230, SE, Brazil; eng.beatrizco@gmail.com (I.B.C.O.-A.); hortencia010@hotmail.com (H.E.P.S.); vieira.ingrid.0405@gmail.com (I.V.F.); ruzeneds@hotmail.com (D.S.R.); 2 CCET—Center for Exact Sciences and Technology, Federal University of Sergipe, São Cristóvão 49107-230, SE, Brazil; maciel.samia@gmail.com; 3 CISAS—Center for Research and Development in Agrifood Systems and Sustainability, Universidade Politécnica de Viana do Castelo, 4900-347 Viana do Castelo, Portugal; joana@estg.ipvc.pt (J.S.);; 4 Estação Zootécnica Nacional, Instituto Nacional de Investigação Agrária e Veterinária (INIAV), 2784-424 Vale de Santarém, Portugal; 5 PROBIO—Graduate Program in Biotechnology, Federal University of Sergipe, São Cristóvão 49107-230, SE, Brazil; 6 PPGPI—Graduate Program in Intellectual Property Science, Federal University of Sergipe, São Cristóvão 49107-230, SE, Brazil

**Keywords:** keratin, biodigestion, pretreatment, feathers, biogas

## Abstract

Keratin is an abundant, recalcitrant structural protein that constitutes the primary component of several animal wastes, particularly chicken feathers. Because of their potential and availability, various technologies, such as anaerobic biodigestion, have been used to degrade keratin and transform feathers into biogas and other value-added products. However, due to their fibrous architecture and rigid structure, keratinous materials are elastic, water-insoluble, and enzymatically resistant, which makes natural degradation difficult. In this sense, before using chicken feathers as feedstock in biodigesters, the keratin in the residue must be cleaved in pretreatment steps. Whether as a single substrate or in co-digestion processes, the keratin breakdown is critical for enhancing biogas production from feathers. In this context, there is growing emphasis on developing pretreatment methods to facilitate protein hydrolysis and digestion, thereby improving biogas generation. To evaluate progress in recycling waste keratin, a bibliometric analysis of original scientific publications on the pretreatment of chicken feathers for anaerobic digestion was conducted using the Scopus database. The findings indicate that researchers apply chicken feathers in processes, including standard biodigestion, co-digestion with food waste, animal manure, and slaughterhouse waste, for biomethane and biohydrogen production. Across the evaluated studies, the pretreatment methods showed notable improvements in feather solubilization and subsequent biogas yield; however, they still encounter key limitations, including ammonia (NH_3_) inhibition, high chemical/reagent costs, and high energy demand.

## 1. Introduction

Due to its high protein content, minimal fat levels, and comparatively low cost, poultry has emerged as a preferred choice among consumers. Surpassing both beef and pork, chicken has become the most popular meat worldwide, accounting for the bulk of the increase in overall meat consumption [1]. Consequently, the poultry slaughter industry now plays a notable role in the generation of residues. According to North and Bell [2], feathers account for 4% to 8% of a chicken’s live weight. Given that the annual global production of chicken meat exceeds 107.5 million metric tons [3], a massive volume of waste feathers is generated, of which only a small fraction is currently recycled [4].

Most feathers are often inadequately treated, typically being buried, dumped, incinerated, or added to landfills. These practices harm the environment, leading to pollution in soil, water, and air, and contributing to human diseases [5]. In the USA, China and Brazil, the leading producers of chicken meat [3], the mismanagement of these residues has become a major environmental issue. Although organic matter, feathers are considered problematic waste and significantly impact the environment because they are about 91% *β*-keratin [6]. Keratin is the primary component in chicken feathers that hinders biodegradation.

Keratin is a structural protein that self-assembles into a hierarchical structure. It extends from nanoscale filament-matrix to macroscale cross-linked polypeptide chains rich in cysteine residues. These chains contain large amounts of disulfide bonds, hydrogen bonds, and hydrophobic interactions [7]. This robust composition and rigid conformation confer elastic properties and water impermeability on keratin materials. Consequently, keratin feathers are difficult to degrade, necessitating prior treatment to disrupt their protein conformation [8]. Pretreatments are designed to break down keratin, thereby facilitating its degradation into soluble peptides and amino acids, which are more readily accessible for a range of applications and processes.

Keratin serves as a valuable protein and raw material across various industries. The extraction of this material represents a critical pathway for enhancing the value and valorization of poultry residues in the fields of healthcare, cosmetics, fuels, food, and other sectors [9]. In scientific research, the use of feathers has been reported in a fermented mixture with soybean by-products to produce a balanced non-poultry livestock feed [10]; combined with tannic acid in a crosslinker layered double hydroxide structure to prepare a functional bio soybean meal-based adhesive [11]; integrated with polyvinyl chloride (PVC) to enhance both acoustic and thermal insulation [12]; utilizing enzymes extracted from feathers for detergent production [13]; and for biochar production [14].

Pretreated keratin waste may also be employed in the production of biogas through the process of anaerobic biodigestion (AB). In the absence of oxygen during anaerobic digestion (AD), keratinous organic matter can be broken down by microorganisms, resulting in the generation of valuable byproducts such as biogas. Biogas is primarily composed of methane (CH_4_), which is commonly utilized as a cooking fuel, and carbon dioxide (CO_2_), along with traces of other gases and water [15]. After removing CO_2_ and other impurities, the gaseous products can be used as cooking gas or for electricity generation [16]. Within the scope of anaerobic digestion, there is also the co-digestion process, which involves the simultaneous digestion of two or more substrates to enhance or optimize biogas production. For example, Gong et al. [17] conducted a pretreatment of feathers, offal, and spoiled chicken meat to diminish hydrogen sulfide (H_2_S) production by approximately 25% to 69% within temperature ranges of 20 to 37 °C, which consequently led to an increase in biogas volume.

In the field of enhancing the value of the poultry production sector from a sustainable perspective, various technological methods have been developed to utilize the energetic potential of feathers. Accordingly, this paper presents a bibliometric review of the Scopus database, which aims to embrace the landscape of scientific research concerning the utilization of pretreated chicken feathers in biogas production as a sustainable alternative to fossil fuels, while simultaneously contributing to the reduction of environmental impact.

Although general reviews exist on poultry waste management and keratin extraction for biomaterials or fertilizers, this study distinguishes itself by focusing on the optimization of chicken feather anaerobic digestion via pretreatment for subsequent biofuel production, thereby fostering a circular bioeconomy within the poultry industry. The novelty of this study rests on three fundamental pillars:

- Novel bibliometric mapping: A quantitative analysis of the historical evolution of publications, international collaboration networks, and high-impact articles alongside their respective journals.
- Comparative analysis of pretreatment technologies: A critical comparison among biological, thermal, and chemical routes regarding energy balances, inhibition mechanisms, and bioproduct formation
- Bridging the lab-to-industry gap: An in-depth discussion on C/N ratio optimization and the necessity of anaerobic co-digestion to balance operating conditions, alongside the technological bottlenecks hindering the transition from bench to pilot scale.

## 2. Materials and Methods

### 2.1. Search Strategy

The search for scientific publications related to the study topic, conducted in the Scopus database, employed a search strategy combining keywords, boolean logical operators, and the characters as follows: (TITLE-ABS-KEY ((“feather*” OR “plumage” OR “plume*”)) AND TITLE-ABS-KEY ((“chicken” OR “hen”)) AND TITLE-ABS-KEY ((“biodigest*” OR “co-digestion*” OR “anaerobic” OR “gas” OR “biogas” OR “energ*” OR “bioenerg*”))) AND (LIMIT-TO (DOCTYPE, “ar”)). The search was conducted within Scopus owing to its comprehensive coverage and extensive index of periodicals across diverse fields, including Engineering, Biotechnology, Chemistry, and Environmental Science. This ensures thorough coverage of literature on the valorization of residues and the energy sector.

The search was conducted in January 2025 and included all work published in the Scopus database up to the end of 2024, in all languages, and limited to original scientific articles.

### 2.2. Screening Strategy

**Figure 1:**
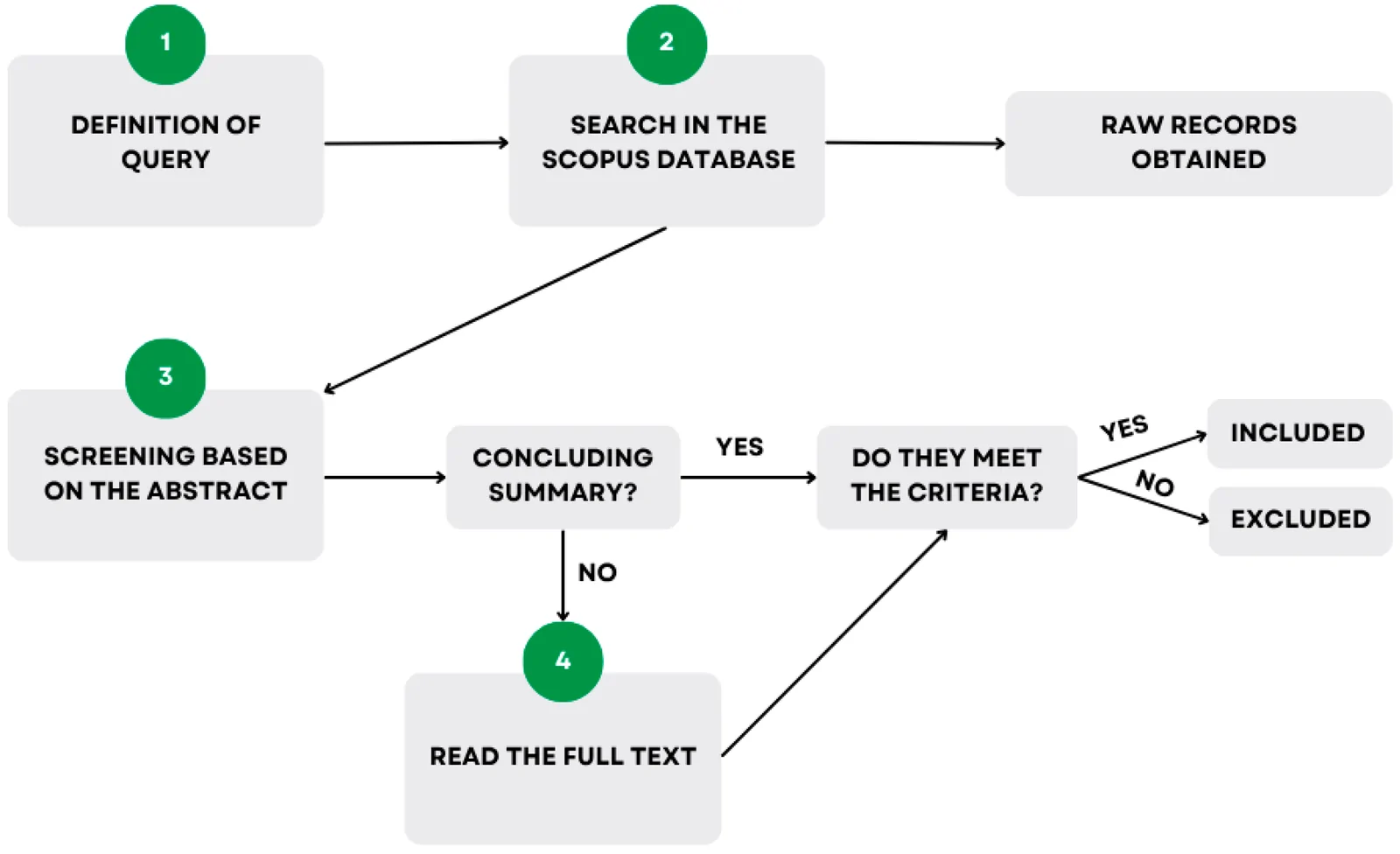
Flowchart of the literature search, screening, and selection process for chicken feather pretreatment in biogas production via anaerobic biodigestion (Scopus database).

The screening of articles followed the methodology illustrated in Figure 1. Initially, the original articles retrieved from the search query (Section 2.1) were screened by title and abstract to confirm their relevance and inclusion in the study. If eligibility could not be confirmed from the abstract alone, the full text was evaluated. To include an article in the present study, the article must satisfy three simultaneous criteria:

- Report some pretreatment method (physical, chemical, biological, or combined) applied to chicken feathers
- Submitted pretreated biomass to the anaerobic digestion process, whether it is mono- or co-digestion
- Evaluate the biogas production process, specifically identifying the product of interest.

Following these criteria, an article was excluded from the study if it focused on the pretreatment of other biomass; if it focused on the characterization of in-nature feathers (work based on non-treated feathers); if it was limited solely to the extraction of keratin to obtain biomaterials, cosmetics, or animal feed without subjecting the biomass to anaerobic biodigestion; or if it directed the hydrolizate to the synthesis of biofertilizer and biomaterials without quantifying biogas.

### 2.3. Bibliometric Maps

The bibliometric network analysis and statistics of retrieved articles were carried out using VOSviewer (version 1.6.18) and tracking citations, authors, institutions, countries, keywords, and journals.

VOSviewer is a software developed to build and visualize bibliometric maps. Contrary to most software generally used for bibliometric mapping, VOSviewer provides special attention to the graphical representation of bibliometric maps [18].

## 3. Results and Discussion

The search performed on the Scopus database resulted in the retrieval of 435 original articles. These articles were carefully examined and excluded if they did not align with the criteria established in Section 2, which required studies to report physical, chemical, biological, or combined pretreatment of chicken feathers, followed by mono- or co-anaerobic digestion, with a focus on biogas production and identification of the target product. After the screening and exclusion of studies that did not meet these criteria, 24 articles were selected for inclusion in the present study.

The works that were not aligned with the studied subject were mainly focused on animal diet, utilizing keratinase to extract keratin from feathers to replace part of the protein in animal feed [19], or the use of chicken feathers and animal bones to produce fertilizers to improve the growth of sunflowers [20]. Additionally, there was research on an anomaly case involving pathogen diseases and deaths in farmed chickens in China [21], production of composite with chicken feathers and other natural fibers to study its acoustic, thermal, mechanical and radiation performance [22] and studies regarding the quality of slaughter chicken meat [23].

The approved articles were categorized by article objective (biodigestion, co-digestion, use of pretreatment, and biofuels production) and subjected to in-depth analysis.

### 3.1. Publishing Trends

**Figure 2:**
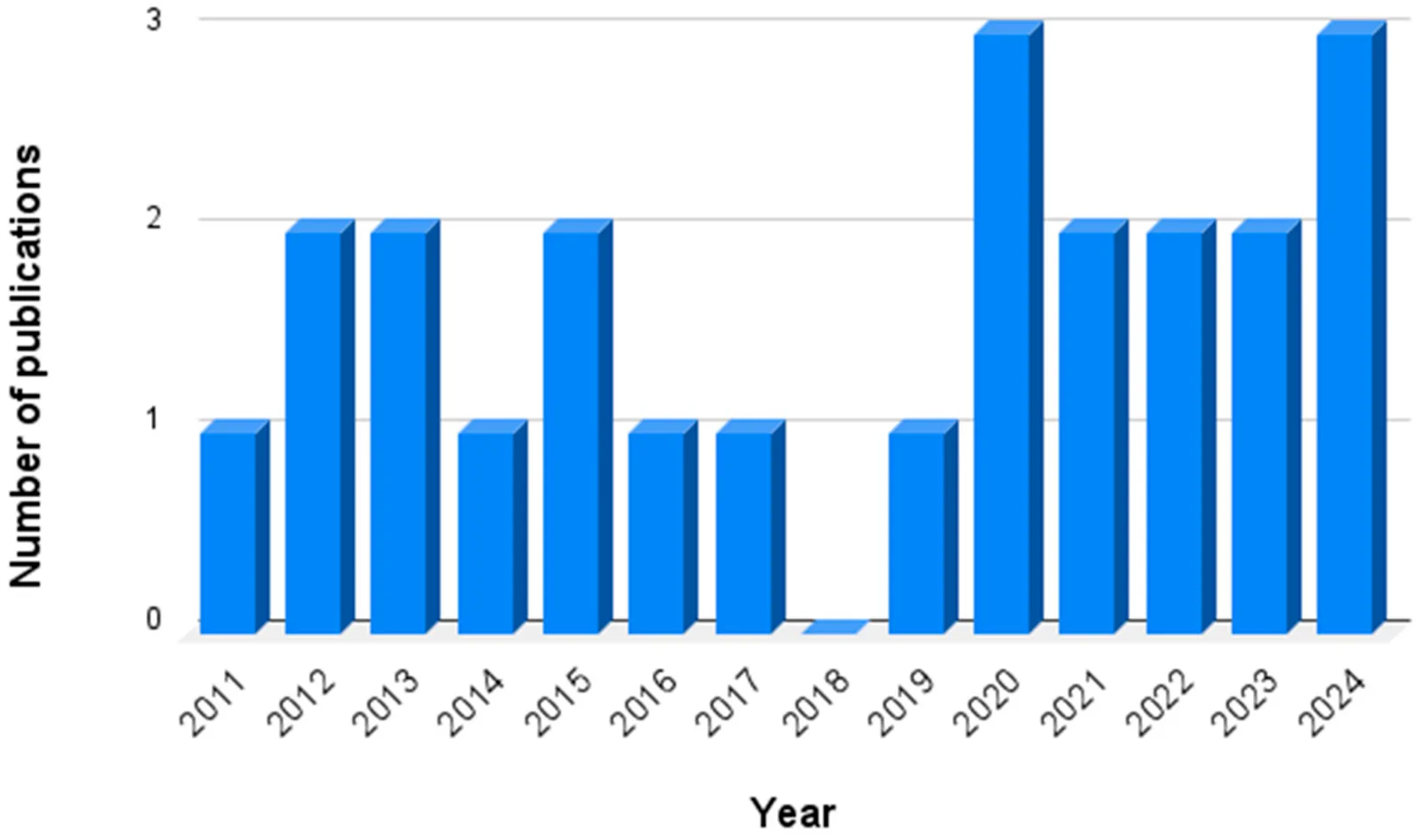
Annual distribution of publications on the anaerobic digestion of pretreated chicken feathers from 2011 to 2024—corresponding to the years in which the eligible publications identified through the screening process were published.

The annual availability of publications related to the anaerobic digestion of pretreated chicken feathers is illustrated in Figure 2. All articles within the scope of the study were identified as published between 2011 and 2024, even though the study did not restrict the search to a particular time period and considered the historical records available within the database. Given the limited volume of research eligible for the study, it is challenging to identify peaks or publication density; nevertheless, there has been a consistent increase in publications since 2019, indicating growing engagement in this sector.

### 3.2. Distribution of Publications by Country and by Institution

Table 1 illustrates the top ten of nineteen identified countries with the highest productivity for biogas production using pretreated feathers.

Sweden leads, accounting for nearly one-fifth of all relevant publications and an impressive total of 192 citations. This position may be explained by Sweden’s investment in renewable and cleaner energy alternatives, especially bioenergy, partly driven by limited domestic fossil fuel resources and its long-term commitment to sustainable economic development. As a result, Sweden’s average number of publications is significantly higher than that of other nations, highlighting its leading contribution to bioenergy research, including biogas production, as shown in this study [24]. Among the most cited countries, South Korea and Portugal also appear with 103 and 50 citations, respectively.

**Figure 3:**
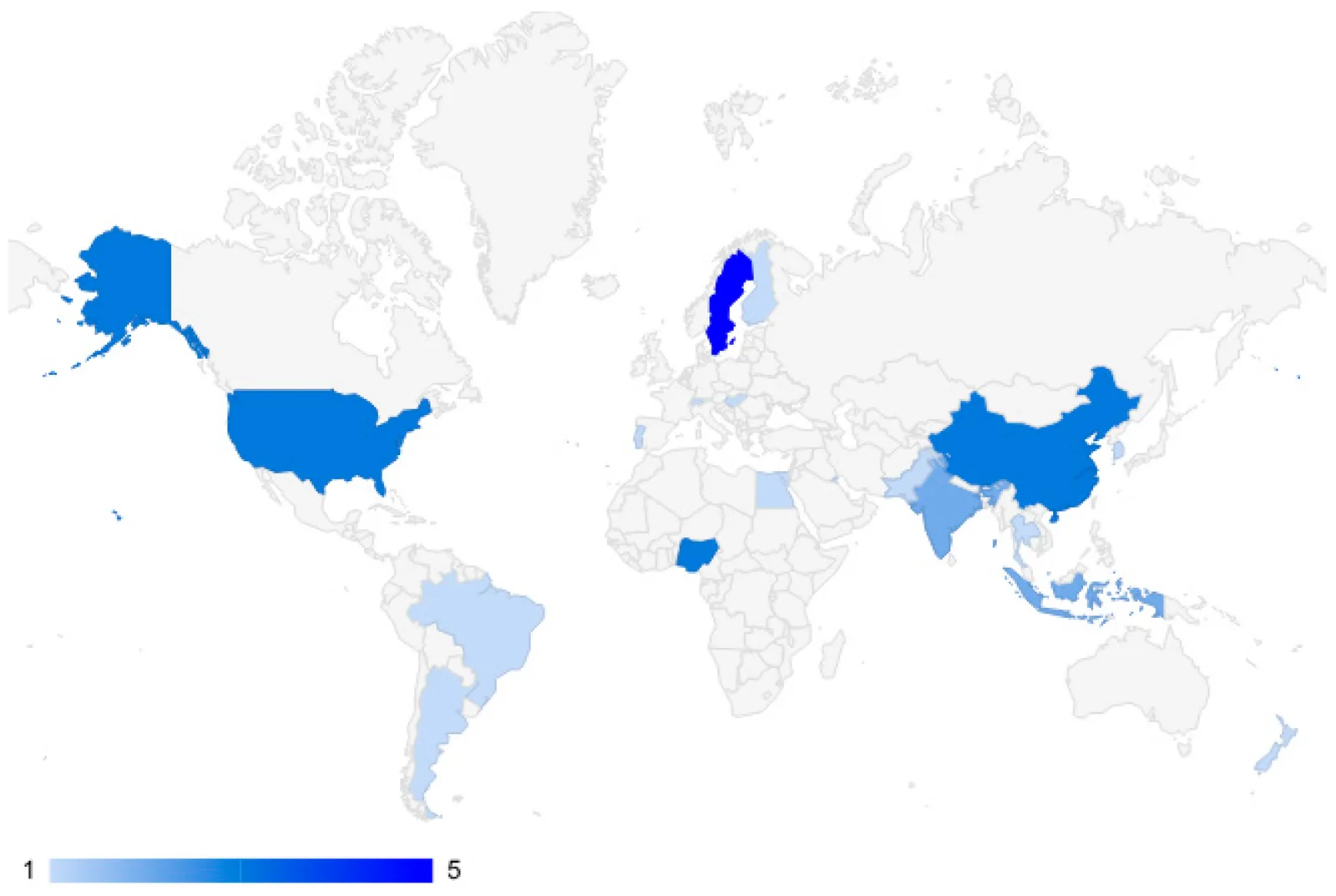
Global distribution of publications on the anaerobic digestion of pretreated chicken feathers. The map shows the geographic distribution of the 19 countries contributing to this research field, with color intensity representing the number of publications from each country.

The geographic distribution of publications from the nineteen involved countries can be seen in the Mundi map in Figure 3. As illustrated in the publications map, research on feather pretreatment for biogas production is mainly concentrated in countries of Asia, North America, and the European Union. The leadership of these research hubs may be attributed to the availability of technological infrastructure, as well as government incentives and policies supporting the valorization of agro-industrial waste.

The United States and China, with three publications each, may reflect their large-scale poultry industries, as they are the world’s two largest poultry producers, and their concerns regarding residue management [3]. This trend also aligns with China’s active policies and initiatives supporting the energy transition and agro-industrial waste valorization [25]. In contrast, countries such as Brazil, Russia, Mexico, Turkey, Argentina, Colombia, and Thailand, which are also among the world’s top ten poultry producers, have contributed only one or no publications to this research area [3]. This suggests a gap in research interest and incentives in this field, despite their strong poultry production, while also highlighting opportunities to expand research in this area.

Another notable observation is Nigeria, which also accounts for three publications, standing out as a relevant contributor in this field despite not being among the largest global poultry producers.

**Figure 4:**
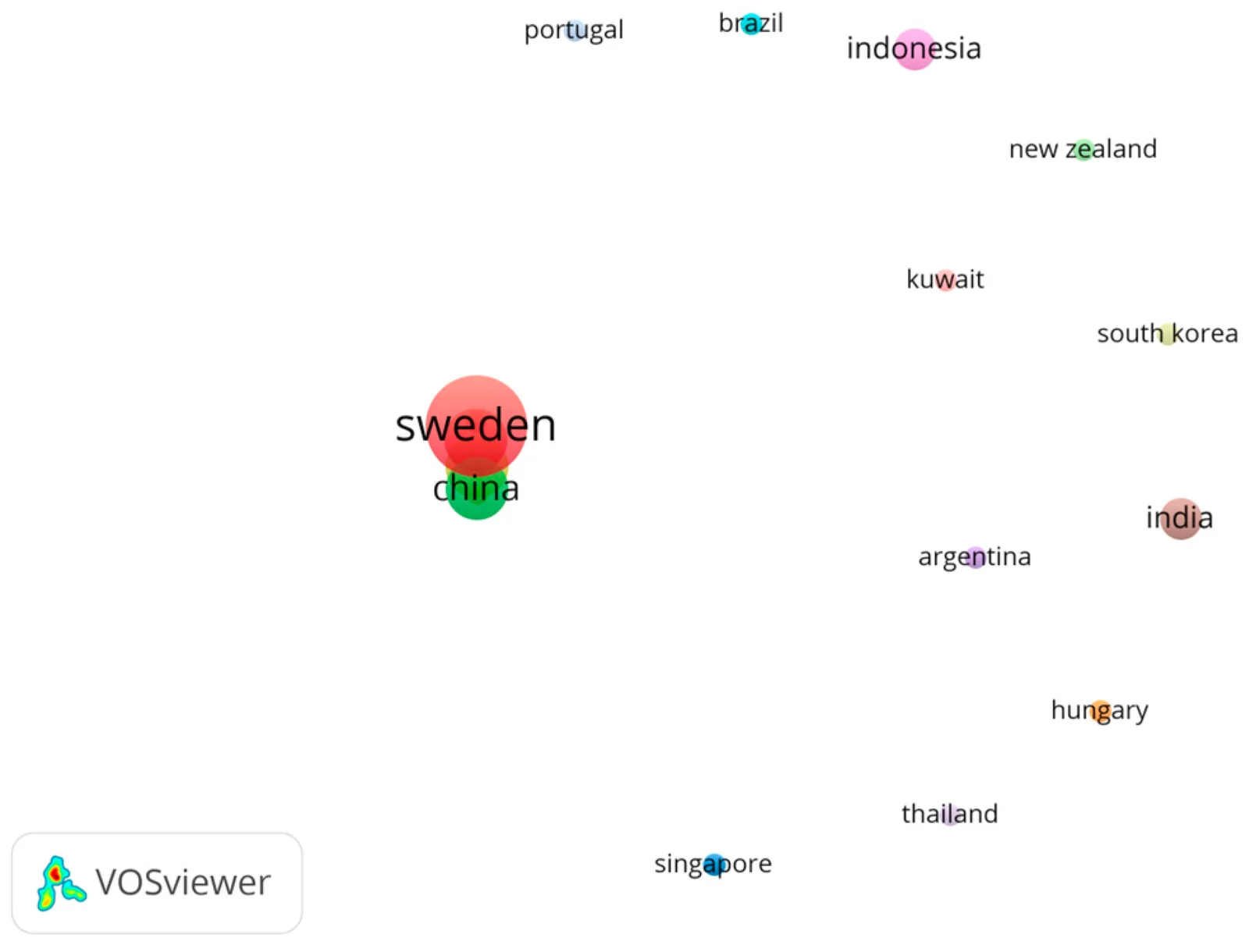
Country collaboration network related to publications on anaerobic digestion of pretreated chicken feathers. The lines indicate co-authorship collaborations among countries.

**Figure 5:**
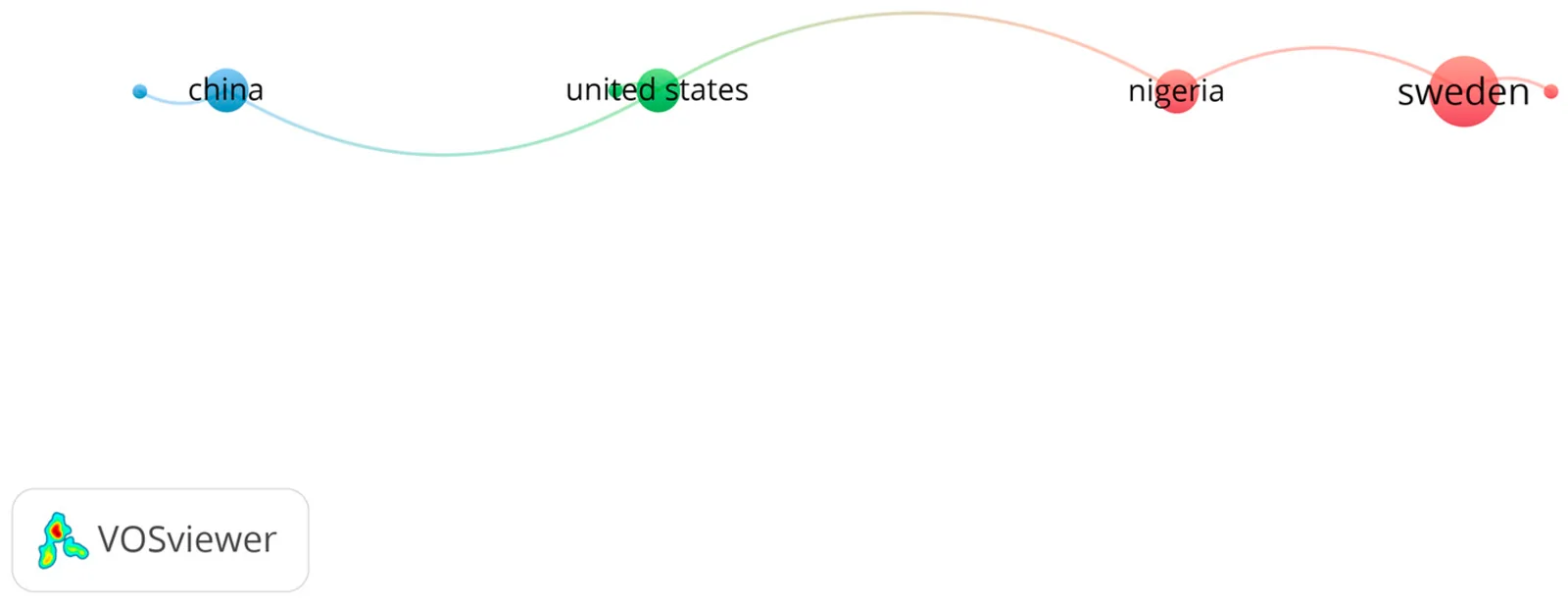
Detailed visualization of the international collaboration network identified among the contributing countries. The network highlights the collaborative links among Sweden, the United States, China, Nigeria, Finland, and Pakistan.

The collaboration map in Figure 4 illustrates the joint efforts among 19 countries, with connecting lines representing collaborative work. Overall, Figure 5 reveals a network characterized by limited connections among six nations: Sweden, the United States, China, Nigeria, Finland, and Pakistan. This scarcity of international collaboration may be attributed to the predominance of bench-scale studies, which typically focus on local demands for agricultural waste treatment rather than dedicated international initiatives. Furthermore, as observed in the evaluated literature, the lack of standardized protocols for keratin residue valorization can hinder international collaboration and technology transfer. To address these challenges, future research should incentivize international partnerships to establish standardized methods, exchange keratinolytic microbial strains, and collaboratively develop pilot-scale bioreactors to drive industrial adoption.

A total of 43 institutions from 19 countries have published articles on the pretreatment of feathers for biogas production. Only two institutions have published more than one article, and just one institution has published more than two articles. Table 2 shows that the institutions with the highest number of publications are from Sweden and South Korea, accounting for six and one publication, respectively, and are responsible for the most cited works. This outcome is consistent with the findings in Table 1.

### 3.3. Keyword Analysis

The analysis of keyword co-occurrence resulted in 495 identified words, of which the authors defined 97 and Scopus defined 459. The total sum of keywords from both groups surpasses the overall total identified, as some of these terms overlap. The study was conducted using all the keywords from both groups, which were analyzed sequentially by searching for synonyms, singular forms, and plurals.

The terms of most occurrence were: “chicken feather”, “animal”, “anaerobic digestion”, and “methane” with 17, 13, 11, and 10 occurrences, respectively. These terms are connected to the terms used for the search and the product from anaerobic digestion: the most cited “chicken feather” is the residue focus of this review; “methane” is related to the product, which is the main component in biogas composition; and “anaerobic digestion” describes the process of methane production. Additionally, the relationships between the most used terms are illustrated in Table 3.

**Figure 6:**
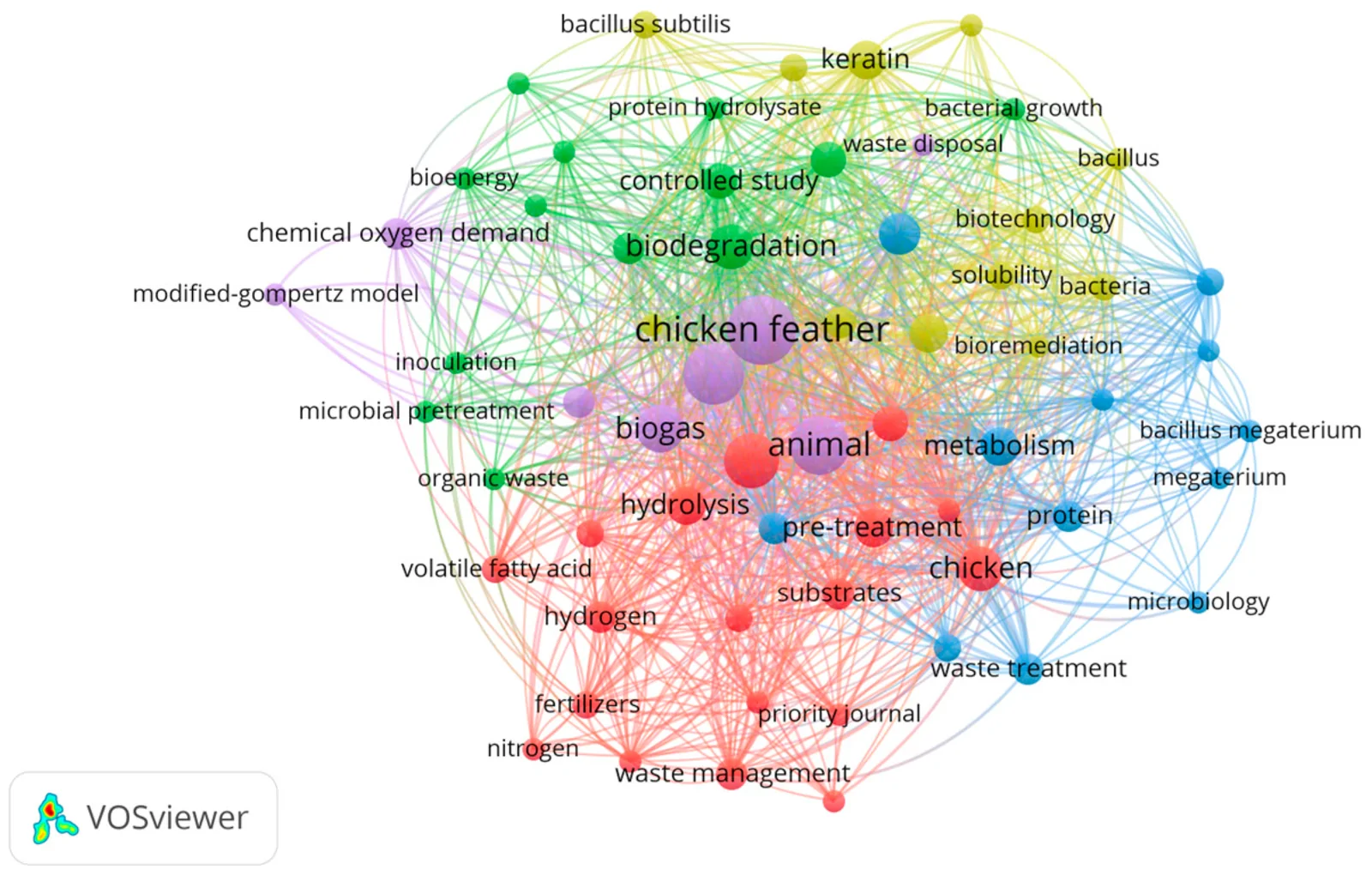
Keyword co-occurrence network generated using VOSviewer. The map shows the main terms associated with the analyzed publications, with larger circles representing keywords with higher occurrence and stronger relationships with other terms. The most prominent keywords include “chicken feather”, “anaerobic digestion”, “chicken”, “methane”, and “animal”.

A diagram illustrating the occurrence of keywords on VOSviewer was created for better visualization of keyword treatment. In the generated map (Figure 6), it is possible to observe that the terms “chicken feather”, “anaerobic digestion”, “chicken”, “methane,” and “animal” have circles of larger diameters, along with other most-cited terms. The more prominent circles represent greater relationships with the other words, indicating that the most cited words are frequently used together.

### 3.4. Most Cited Articles

The quantitative evaluation of the impact and influence of the mapped publications enables the identification of the conceptual foundations that guide the state of the art in chicken feather valorization. Table 4 presents the most highly cited publications in the collected dataset, correlating citation counts with the journals in which they were published and their respective Journal Citation Reports (JCR) Impact Factors (IF).

The analysis of Table 4 indicates that scientific production on poultry waste treatment is predominantly concentrated in high-impact journals specializing in biotechnology, environmental engineering, and waste management, particularly Renewable Energy (IF-JCR 9.1), Bioresource Technology (IF-JCR 9.0), and Waste Management (IF-JCR 7.1). This concentration underscores the interdisciplinary nature of research on poultry waste valorization and its strong connection with sustainable bioprocessing and waste management.

Among the most influential contributions, Forgács et al. [26] and Patinvoh et al. [27] investigated biological and enzymatic pretreatments, receiving the highest citation counts within the dataset. Their studies demonstrated the potential of keratinases to overcome the recalcitrant structure of feathers while reducing the reliance on harsh chemicals and energy-intensive processing. In contrast, Costa et al. [28] and Strong and Gapes [29] provided important contributions to understanding the molecular and process-level limitations of feather degradation. Costa et al. [28] identified the conversion of soluble products, rather than the hydrolysis rate itself, as a critical bottleneck, whereas Strong and Gapes [29] investigated the thermal and oxidative conditions required to promote degradation while minimizing the loss of biodegradable carbon.

**Table 4:** Top ten most-cited articles on chicken feather pretreatment for anaerobic digestion indexed in the Scopus database.

Article Title	Year	TC	Journal	IF	Ref.
Biological treatment of chicken feather waste for improved biogas production	2011	62	Journal of Environmental Sciences	6.5	[26]
Biological Pretreatment of Chicken Feather and Biogas Production from Total Broth	2016	53	Applied Biochemistry and Biotechnology	3.3	[27]
Effects of pre-treatment and bioaugmentation strategies on the anaerobic digestion of chicken feathers	2012	50	Bioresource Technology	9.0	[28]
Pretreatment of chicken feather waste for improved biogas production	2013	38	Applied Biochemistry and Biotechnology	3.3	[30]
Thermal and thermochemical pre-treatment of four waste residues and the effect on acetic acid production and methane synthesis	2012	30	Waste Management	7.1	[29]
Anaerobic co-digestion of swine manure and chicken feathers: Effects of manure maturation and microbial pretreatment of feathers on methane production	2020	29	Renewable Energy	9.1	[31]
Dry Anaerobic Co-Digestion of Citrus Wastes with Keratin and Lignocellulosic Wastes: Batch And Continuous Processes	2020	21	Waste and Biomass Valorization	2.8	[32]
The keratinolytic bacteria Bacillus cytotoxicus as a source of novel proteases and feather protein hydrolysates with antioxidant activities	2021	20	Journal of Genetic Engineering and Biotechnology	3.4	[33]
Using slaughterhouse waste in a biochemical-based biorefinery–results from pilot scale tests	2017	18	Environmental Technology (UK)	2.0	[34]
Feather Waste Recycling for Biogas Production	2015	15	Waste and Biomass Valorization	2.8	[35]

TC: total citations; IF: Impact Factor from Journal Citation Reports (JCR); Ref.: reference.

### 3.5. Publication Analysis and Hot Issues of Selected Literature

**Figure 7:**
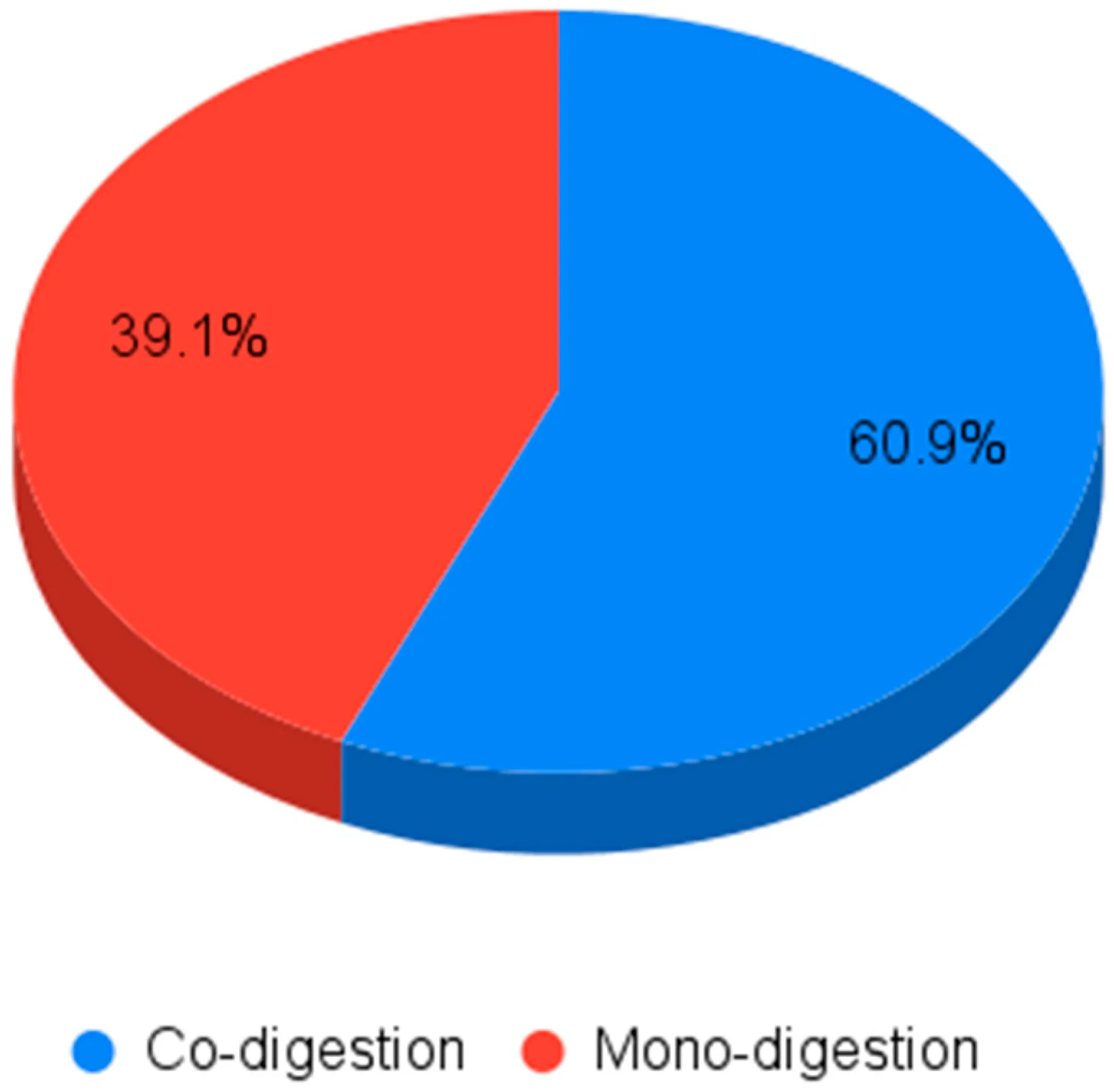
Distribution of the studies on biodigestion of pretreated chicken feathers according to the anaerobic digestion strategy.

Among the selected studies, it was observed that some focused solely on the pretreatment of feathers to optimize biogas production, while others included feather pretreatment along with additional strategies, such as co-digestion with food or agro-industrial waste. The analysis of the articles indicates that anaerobic co-digestion (60.9%) is predominant. In this process, the reuse of food waste, animal manure, and/or slaughterhouse waste in co-digestion with chicken feathers was identified. The studies were initially grouped into anaerobic digestion and co-digestion, as shown in Figure 7.

**Figure 8:**
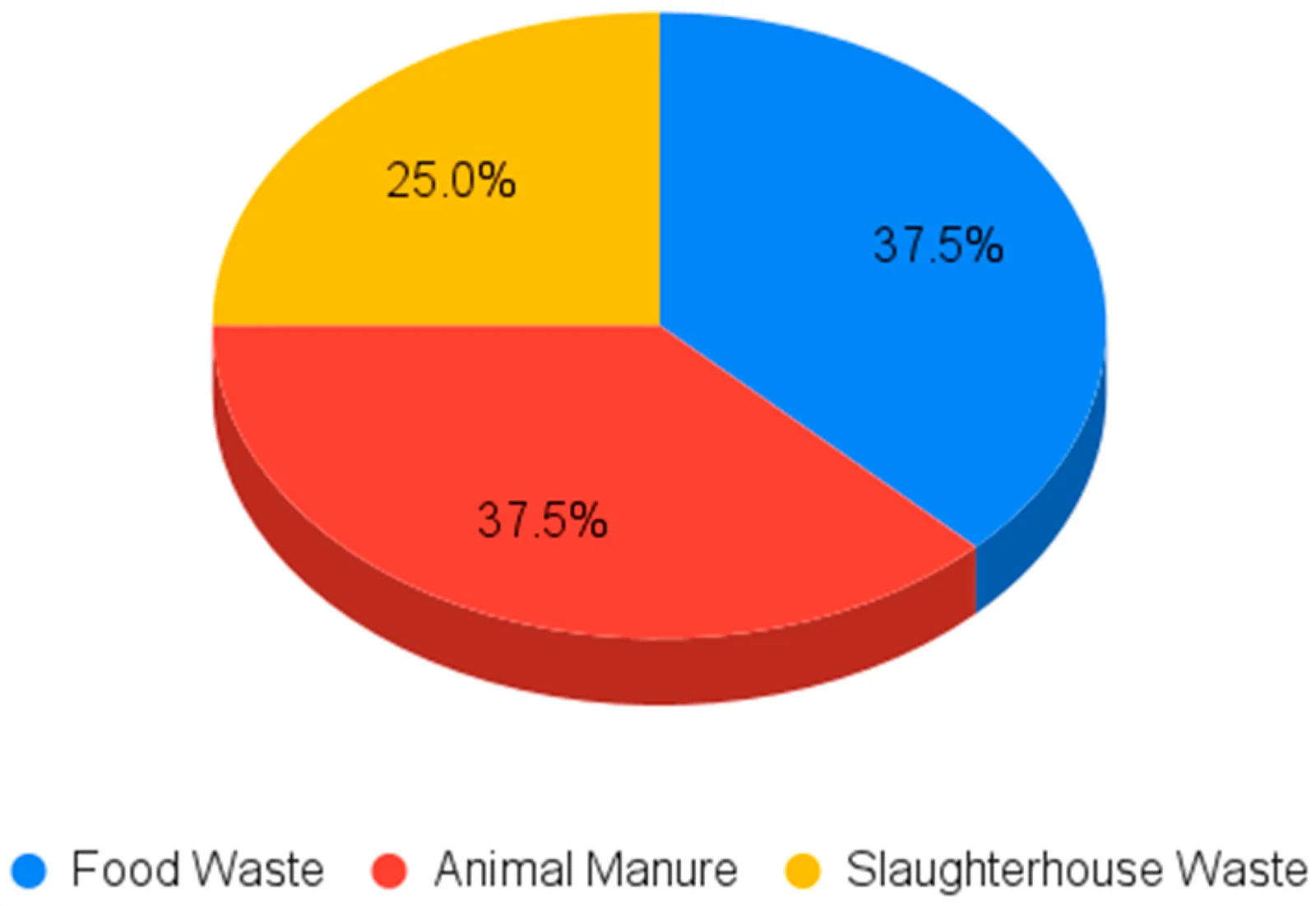
Distribution of co-substrates used in the anaerobic co-digestion of pretreated chicken feathers.

The data presented in Figure 7 reflect the inherent constraints of feather anaerobic digestion. Because feather structures are predominantly protein, mono-digestion (39.1%) often leads to a low carbon-to-nitrogen (C/N) ratio and ammonia accumulation, which inhibits methanogenic archaea [36]. Consequently, most studies have focused on co-digestion (60.9%) as an effective strategy to balance nutrient availability within the bioreactor and ensure process stability. Furthermore, the co-substrates illustrated in Figure 8 align with logistical feasibility criteria: food waste (37.5%) provides carbon to rebalance the organic load and C/N ratio [36]; animal manure (37.5%) serves as an active inoculum while ensuring pH buffering capacity [34]; and slaughterhouse waste (25%) enables on-site energy integration within industrial hubs, minimizing transportation costs and valorizing the entire waste stream of the poultry industry.

#### 3.5.1. Mono-Digestion

Anaerobic mono-digestion (found in 39.1% of studies) is a process in which a single type of organic matter is degraded in an anaerobic environment by specific microorganisms, including fermentative bacteria, acetogenic bacteria, and methanogenic archaea [37]. Subsequently, methanogenic archaea metabolize the degraded constituents to generate biogas, which is mainly composed of methane, carbon dioxide, water vapor, hydrogen sulfide, hydrogen gas, and various other gases [38].

Studies such as that of Patinvoh et al. [27] show how feathers, especially pretreated ones, can have a high potential for biogas generation. In their work, biological treatment of chicken feathers using a *Bacillus* strain was employed, which proved to be highly productive, with approximately 75% of the feathers converted into soluble crude protein after 8 days of degradation. Furthermore, the evaluated pretreatment increased methane yield by 124% compared to the digestion of untreated feathers.

In the research conducted by El Salamony [39], the authors examined the utilization of chicken feathers for methane gas production via anaerobic biodigestion. To improve results, the feathers underwent a biological treatment with *Bacillus subtilis* DB100, resulting in 86% removal of DBO. Subsequently, the methane generation process yielded an electrical potential of 375 mV with a current of 0.52 mA, thereby demonstrating that feathers’ hydrolyzers can be employed for energy generation.

#### 3.5.2. Co-Digestion

Anaerobic co-digestion is the simultaneous digestion of two or more substrates, carried out to evaluate the effect of substrate mixing on improving biogas production. Among the studies that presented co-digestion, the co-digestion of feathers with food waste (37.5%) and animal manure (37.5%) stands out, followed by slaughterhouse waste (25%), as shown in Figure 8.

##### Food Waste

In the study conducted by Saba et al. [40], chicken feather pretreatment was performed utilizing the bacterium *Pseudomonas aeruginosa*, subsequently followed by co-digestion with rice husk and vegetable residues, which significantly increased biogas production compared to the experiment involving untreated feathers. Furthermore, the researchers noted that extending the pretreatment to other substrate residues yielded further improvements in biogas production.

Sumardiono et al. [36] investigated the co-digestion of chicken feathers and coffee pulp post a delignification pretreatment. This approach facilitated the microbial decomposition of the substrate, enhancing its conversion into biogas. The study also evaluated the influence of the carbon-to-nitrogen (C/N) ratio and the percentage of total solids on biogas yield. The results indicated an optimal production with a C/N ratio of 25 g/g and a total solids concentration of 25%.

Furthermore, Kannan et al. [41] utilized different proportions of banana stem juice and chicken feathers to enhance biogas production, resulting in an optimization of biogas production by 161%.

##### Animal Manure

Manures and feathers are among the most abundant and environmentally challenging wastes produced by the livestock and poultry industries [42]. In this context, Schommer et al. [31] proposed evaluating the influence of the pretreatment step by comparing the co-digestion of fresh and matured swine manures with feathers, either submitted to or not submitted to microbial pretreatment using keratinolytic bacteria, which are organisms that produce proteolytic enzymes (keratinase) able to digest insoluble keratin [43]. As a result, the authors reported that when using untreated feathers and fresh manure, only a small volume of biogas could be produced. However, with pretreatment, the mixture of residues increased methane yield, demonstrating the potential of feathers when appropriately prepared.

Other authors, such as Yusuf et al. [44], also observed that treated chicken feathers are more easily degraded by microorganisms, enabling them to break down the long chains of keratin and yield more biogas in a shorter time when co-digested with animal manure and/or slaughterhouse waste.

##### Food Waste and Animal Manure

Mézes and Tamás [35] investigated biogas production through the co-digestion of corn silage, bovine manure, and slaughter waste, which included enzyme-pretreated feathers in varying proportions (5%, 10%, 15%, and 20%). The highest methane yield of 0.37 ± 0.16 Nm^3^/kg Volatile Solids (VS) was achieved with the mixture containing 5% pretreated feathers. This peak indicates a limit to the amount of methane that can be absorbed from the feathers. Moreover, since 5% was the lowest proportion tested, further evaluation of lower percentages is necessary to confirm the validity of this result.

In addition to identifying the critical percentages of materials, it is also essential to investigate the mixing ratios between substrates in the co-digestion process. Then, Patinvoh et al. [32] investigated different mixing ratios for the co-digestion of citric residues with chicken feather, wheat straw, and manure bedded with straw. The best performance was observed at a mixing ratio of 1:1:6:0. This specific mixture produced 238 mL/g VS (volatile solids) of methane, representing a 14% increase compared to the digestion of the individual components.

In another study, the authors pretreated chicken feathers using physical, biological, and chemical methods, reporting that the pretreatment accelerates the process of feather degradation by up to three times. When chicken feathers were pretreated with Ca(OH)_2_ and co-digested with cow dung, digestion improved biogas production [44].

##### Slaughterhouse Waste

The slaughterhouse wastes are often sent to pig farms for use as feed. However, if not managed properly, these wastes can lead to soil and water pollution, as is typically the case. In the study by Schwede et al. [34], the authors proposed a biorefinery concept utilizing 2,3-butanediol fermentation, acetone-butanol fermentation, and methane fermentation, using slaughterhouse waste, chicken manure (including pretreated chicken feathers), and straw as substrates. In the experiment, an increase in pH resulted primarily in the formation of acidic compounds such as butyrate, propionate, γ-aminobutyrate, and valerate. Meanwhile, the residual liquid from the bioreactor displayed a high yield of biogas. The observed results indicate that integrating biorefinery with biogas recovery through anaerobic digestion of protein-rich wastes could be an effective approach for simultaneously producing usable chemicals and bioenergy.

Strong and Gapes [29] investigated the co-digestion of thermally pretreated coal, kraft pulp solids, chicken processing waste, and chicken feathers. The authors reported that when subjected to an inert or oxidative atmosphere at temperatures up to 140 °C, feathers more than tripled methane yield. However, at temperatures above 200 °C, the oxidative atmosphere favored acetic acid production and significantly decreased methane yield. This indicates that the thermal-chemical reactions and the destructive nature of oxidative pretreatment resulted in less carbon available for methane generation. Therefore, while thermal pretreatment promotes a more rapid and greater conversion of residual carbon to methane, thermal-chemical treatment could lead to the rapid breakdown of solid residues and the formation of more desirable compounds.

#### 3.5.3. Types of Pretreatments Used in Feathers

**Figure 9:**
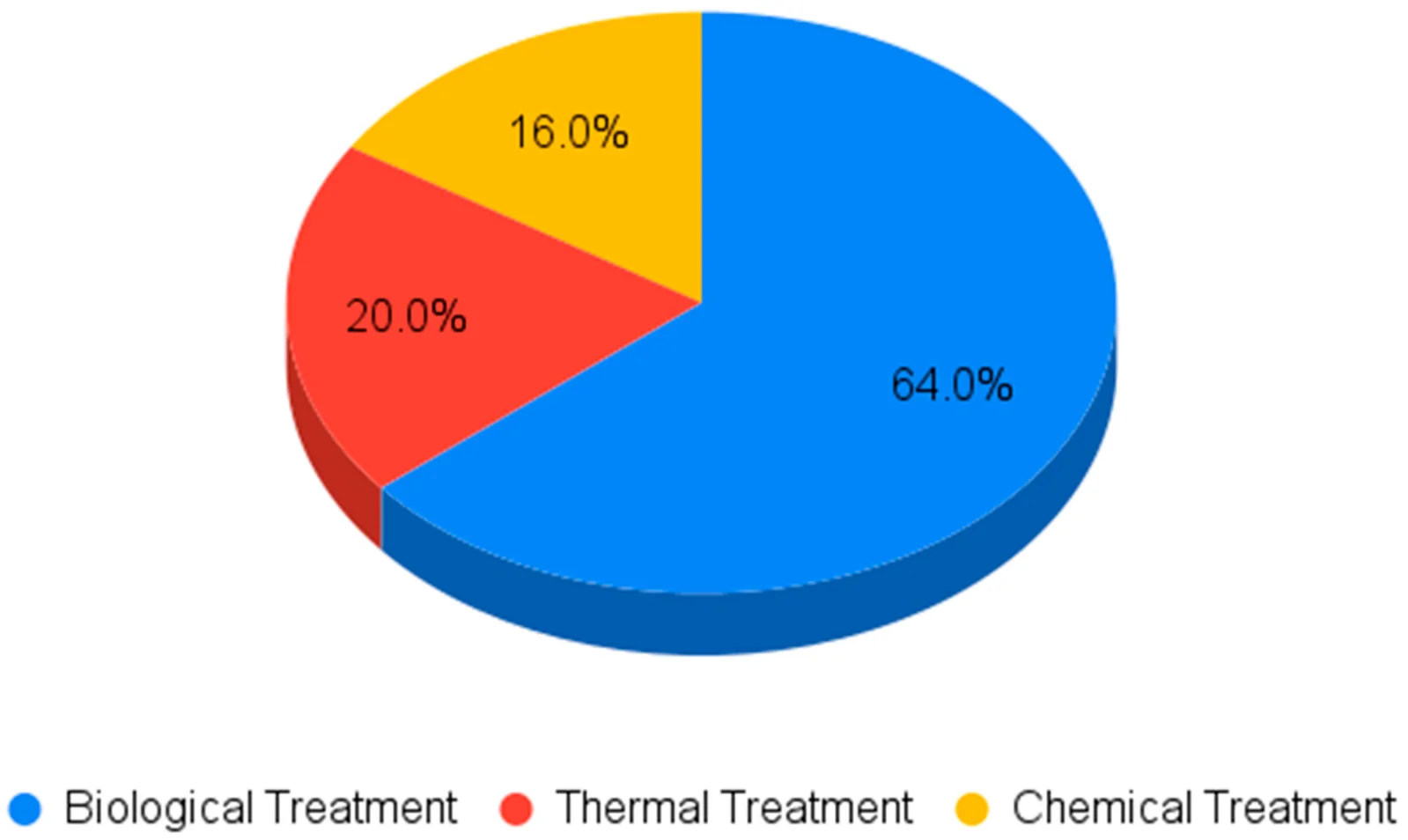
Distribution of pretreatment methods applied to chicken feathers before anaerobic digestion.

The articles were categorized according to the type of treatment employed, with several studies utilizing multiple experimental approaches. The main reason for incorporating multiple pretreatments is to compare methods and determine the most effective approach for the specific substrate under analysis. The distribution of these treatments and their prevalence within the overall body of research are illustrated in Figure 9.

The data presented in Figure 9 show that biological routes are the most widely investigated (64%), driven by mild operating conditions, low energy consumption, and the absence of toxic effluents. Thermal processes (20%) are effective in disrupting the crystalline structure of keratin, but they require high energy inputs and generate greenhouse gas emissions. Finally, chemical treatments account for only 16% of the studies; although they provide faster reaction rates, they pose severe operational challenges, including the formation of inhibitory compounds and high acid/base consumption, which increases operating costs and limits their applicability at industrial scale.

##### Biological Treatment

For the biological treatment of chicken feathers, chicken manure is often used because it naturally contains keratinolytic bacteria, such as those in the genus *Bacillus*. A notable example is the study by El Salamony [39], who utilized a strain of *Bacillus subtilis* to accelerate feather degradation, achieving a significant degradation rate of 80%.

Li et al. [45] also used strains from the genus *Bacillus*, along with *Chryseobacterium*, *Lysobacter, Brevibacillus*, and *Stenotrophomonas* isolated from poultry farm soil. Using these microorganisms, they achieved a feather degradation rate of about 58% within 120 h of cultivation. In contrast, Revankar et al. [46] employed a strain of *Bacillus velezensis* isolated from forest soil in the Western Ghats of Karnataka, a mountain range on the western coast of the Indian peninsula. Through process optimization, they attained a 374% increase in keratinase production, which are specialized proteases capable of hydrolyzing keratin particles.

##### Thermal Treatment

Regarding thermal treatments, Strong and Gapes et al. [29] submitted the feather residues to 70, 140, and 200 °C under inert or oxidative atmosphere conditions. The inert atmosphere utilized nitrogen as a controlled environment to prevent undesired chemical reactions, while the oxidative atmosphere contained sufficient oxygen to promote oxidation. Methane production tripled when feathers were treated at 140 °C in both atmospheres; however, yields decreased significantly following oxidative treatment at 200 °C. These results establish a critical temperature threshold for effective thermal pretreatment. In contrast, Forgács et al. [26] employed different methodologies, including thermal treatment at 120 °C for 10 min, alkaline enzymatic hydrolysis at 55 °C for 0, 2, or 24 h, and a combination of both techniques. The authors revealed that the enzymatic treatment achieved a 122% improvement in methane production compared to untreated feathers, while the other treatmhent showed an increase of 11–50%. Then, the authors concluded that the enzymatic treatment yields the most effective results.

##### Chemical Treatment

Chemical pretreatments are predominantly based on the use of alkalis (NaOH and Ca(OH)_2_) or strong acids (H_2_SO_4_), which act by disrupting disulfide bonds and cleaving peptide linkages in keratin, achieving volatile solids solubilization rates close to 90% [28,36]. However, their transition to industrial scale faces major bottlenecks: high consumption of chemical reagents and the need for downstream neutralization, which increase operational costs; elevated digestate salinity, which can inhibit the metabolic activity of methanogenic archaea; and equipment corrosion risks in industrial bioreactors and pipelines.

In the study by Sumardiono et al. [36], feathers were immersed in a 5% NaOH solution for 24 h. After this period, the solution was neutralized by adding water and sulfuric acid, adjusting the pH to 7. Subsequently, the treated residues were transferred to the biodigestor. According to the authors, this process accelerated substrate decomposition and significantly increased biogas production.

Costa et al. [28] used a combination of treatments for feather degradation. First, they employed a biological process using bacteria *Fervidobacterium pennivorans*, known for degrading keratin. This initial step resulted in a degradation rate between 45 and 64%. Then, a chemical treatment with a solution of 2 g NaOH was applied, increasing the degradation rate to 96%.

#### 3.5.4. Pretreatment Comparisons

Table 5 presents a comparative assessment of the technologies reported in the analyzed studies. This analysis demonstrates that selecting an optimal method extends beyond solubilization efficiency alone. Industrial feasibility depends on balancing hydrolysis kinetics and methanogenic stability within the digester.

Thermal and thermochemical methods accelerate the hydrolysis process; however, they require high energy consumption, carry the risk of process inhibition from excessive free ammonia, and can lead to carbon degradation at temperatures exceeding 200 °C. In contrast, biological and enzymatic routes operate under mild conditions without generating secondary effluents, though they require preliminary stages with longer retention times than other pretreatment approaches.

Given these constraints, chicken feather valorization should not be conducted via mono-digestion, since the rate-limiting factor is the conversion of biomass into biogas rather than the hydrolysis step itself. To achieve optimal performance, pretreatment should be followed by co-digestion with carbon-rich biomass to balance the C/N ratio, alongside integrating waste heat recovery from poultry slaughterhouses to ensure a favorable net energy balance.

#### 3.5.5. Types of Fuels Produced

**Figure 10:**
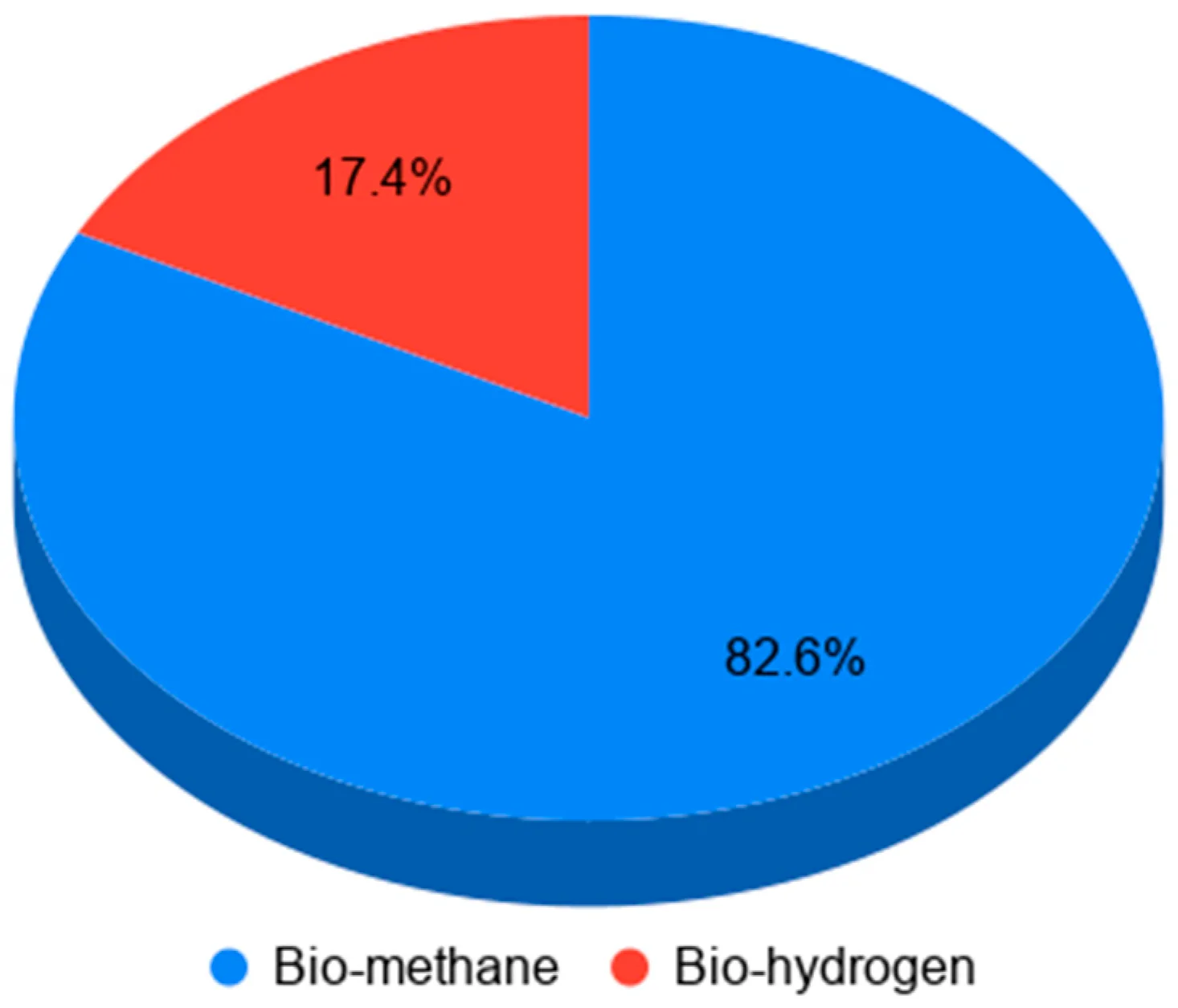
Distribution of the main products obtained from the anaerobic digestion of pretreated chicken feathers.

Biomethane and biohydrogen gases can be produced through anaerobic biodigestion. Biohydrogen is generated in the early stages of the biodigestion process. To maximize biohydrogen production, the process is stopped before the methanogenesis phase, because during this stage, methanogenic archaea would consume the available hydrogen to produce biomethane [34]. Accordingly, an analysis of the research focus across the identified studies was conducted, resulting in Figure 10, which shows the share of biomethane and biohydrogen production.

The data in Figure 10 show that biomethane is the focus of 82.6% of the analyzed studies, reflecting the greater technological maturity of anaerobic methanogenesis and the process’s relative simplicity, which requires fewer operational interventions and follows a pathway closer to natural biodegradation [26,28,30]. In contrast, biohydrogen accounts for only 17.4% of the studies and remains predominantly at the laboratory scale, despite its higher energy content per unit mass compared with biomethane. This lower representation may be attributed to the greater operational complexity of biological hydrogen production, particularly the need for stringent control of pH and temperature to favor hydrogen-producing pathways and suppress subsequent microbial processes that consume H_2_ and convert it into methane [34,47].

##### Biomethane

Biomethane is one of the main gases that make up biogas, which is a mixture of gases produced during anaerobic biodigestion. The refinement of biomethane, after removing CO_2_, water vapor, and hydrogen sulfide, has more energy potential than raw biogas. In this context, research focused on quantifying biomethane is essential in this field.

The work of Costa et al. [28] studied the effect of pretreatments using lime and NaOH or bioaugmented with *F. pennivorans*. They found that the biomethane potential of pretreated feather waste was lower (25 to 105 L CH_4_ kg^−1^ VS—liters of methane per kilogram of volatile solids) compared to the test with raw waste (123 ± 3 L CH_4_ kg^−1^ VS), indicating that none of the tested strategies improved methane production from feather waste. Pretreatment with 0.2 g NaOH g^−1^ resulted in the lowest methane production despite exhibiting the highest solubilization yields (96% compared to 48% in raw feathers) and accumulating high chemical oxygen demand. Therefore, the authors concluded that hydrolysis of the material was not the limiting factor in biogas production, but rather the conversion of soluble organic matter into methane, possibly due to metabolite accumulation that may have inhibited the methanogenic phase.

Additionally, Schommer et al. [31] studied the co-digestion of pig manure with treated and untreated chicken feathers, resulting in a production of 160–190 L CH_4_/kg VS when pig manure was co-digested with treated feathers, which is 15–25% higher compared to the use of untreated feathers.

##### Biohydrogen

Biohydrogen is the H_2_ produced from biomass through thermochemical conversion, such as gasification, or biological processes like anaerobic digestion. Both processes have garnered significant attention as they can utilize waste biomass as feedstock.

In anaerobic digestion, the products are mainly acids and biogas, which include hydrogen as one of the gases in their basic composition [48]. The process can then be adjusted to achieve a configuration that favors hydrogen formation, creating an environment with conditions favorable to the growth and activity of acetogenic bacteria, which convert fatty acids, alcohols, and acids into acetate, CO_2_, and H_2_ [49]. According to Dinesh et al. [50], optimal conditions for H_2_ generation occur at neutral pH and temperatures between 35 and 60 °C, depending on the selected raw material. The study by Dinesh et al. used thermally pretreated chicken feathers to produce biohydrogen via anaerobic digestion, creating an environment favorable to the growth of acetogenic bacteria, achieving a maximum hydrogen yield of 140 mL/L after degradation from *Pseudomonas aeruginosa* at pH 11.

**Table 5:** Analytical comparison among chicken feather pretreatment methods: degradation efficiency, operating conditions, biogas yield, cost and energy balance, environmental risks, and technological bottlenecks.

Pretreatment	Keratin Degradation Mechanism	Operating Conditions and Typical Agents	Increase in Biogas Yield	Energy Balance and Costs	Environmental Risks and Operational Bottlenecks	Ref.
Biological/Enzymatic	Enzymatic reduction of disulfide bonds (-S-S-) and peptide bonds via bacterial keratinases and proteases.	Use of *Bacillus* strains (*B. subtilis*, *B. licheniformis*, *B. cytotoxicus*) or fungi (*Coprinopsis* sp.) in submerged fermentation, 30–55 °C, pH 7–9, 2–7 days.	Significant gains in digestibility, reaching a +100% increase in CH_4_ production	Highly favorable balance: it operates under mild temperature conditions, without the need for intense heating. Costs are linked to the cultivation/enzyme isolation time.	Slow kinetics (long retention times); microbial sensitivity to temperature and pH fluctuations; need for biosafety control of the strains.	[26,27,30,31,33,40,50,51]
Thermal/Hydrothermal	Physical denaturation of the protein structure and breakage of disulfide bonds under heat and pressure.	Autoclaving and moist heat treatment (120–180 °C) for short periods of 15–60 min.	Increase in CH_4_ of 11–50% at 120 °C (10 min). Drop in yield at 200 °C under O_2_ (oxidative destruction of carbon).	Unfavorable energy balance: high primary energy consumption (steam/electricity). High cost if waste heat is not recovered.	Potential formation of recalcitrant Maillard reaction compounds at temperatures above 140 °C; high operational carbon footprint.	[26,29,47]
Chemical	Chemical cleavage of polypeptide chains by acid/alkaline hydrolysis.	Concentrated acids and bases (NaOH, KOH, H_2_SO_4_) under stirring.	Increase of 40–53% in biogas compared to untreated trials.	High operating cost due to the consumption of chemical etching reagents and neutralizing agents.	Generation of high-salinity effluents, potential residual toxicity to methanogenic archaea, and risk of reactor corrosion.	[36,52]
Combined/Thermochemical/Biochemical	Molecular breakdown via heat or chemical reagents, followed by enzymatic biochemical conversion.	Treatment with NaOH or Ca(OH) at 90 °C, 1.27 bar; bioaugmentation (*F. pennivorans,* 65 °C); cascaded enzymatic/thermal hydrolysis (80 °C → 50 °C → 37 °C).	When applied individually, the treatments increased solubilization but reduced CH_4_ production (25–105 L/kg VS vs. 123 L/kg VS in the control). In the integrated biorefinery, biogas production increased by over 450% compared to the raw substrate alone.	Neutral balance, positive when integrated into pilot-scale biorefinery plants with thermal integration.	High operational complexity across multiple stages and accumulated costs for equipment and chemical control.	[28,29,34]

## 4. Future Perspectives and Recommendations

The techno-economic feasibility of converting chicken feathers into biogas at an industrial scale relies on overcoming the thermodynamic, kinetic, and operational bottlenecks identified in the literature. Evaluating these studies, alongside the advantages and limitations of each pretreatment method, clearly underscores the need for future research to refine and optimize these technologies.

Biological and Enzymatic Treatments: The advantages of these methods include mild operating conditions (30–55 °C) at atmospheric pressure, eliminating the need for chemical reagents and lowering the overall carbon footprint. However, reaction kinetics remain slow, requiring retention times of several days, and enzyme purification is costly, with the risk of biological activity loss due to fluctuations in pH, temperature, or microbial contamination. Therefore, future research should focus on developing robust microbial consortia, as well as isolating cost-effective keratinolytic strains from local agro-industrial residues, such as animal manure and other unexplored sources.

Thermal and Hydrothemal treatment: Among the primary advantages of this pretreatment method are the rapid structural denaturation and keratin solubilization achieved within short processing times (10–60 min), and the complete inactivation of pathogens present in the residue. However, the process entails an unfavorable energy balance, characterized by high electricity consumption, a high carbon footprint, and the potential formation of recalcitrant by-products.

Therefore, investigating the thermal integration of waste heat from poultry processing facilities is recommended to optimize energy efficiency, while maintaining operating temperatures within 130–150 °C to prevent the thermal degradation of biodegradable substrate.

Chemical and thermochemical treatments: The primary advantages of this pretreatment method include a high volatile solids solubilization rate (approximately 90%) and the rapid breakdown of recalcitrant crystalline fractions, besides the potential valorization of recovered keratin for applications in the pharmaceutical and biomaterials sectors. Despite these benefits, chemical routes are costly due to the reagent consumption, generate high-salinity effluents, and carry the risk of inhibiting methanogenic archaea due to potential residual acidification. Therefore, future research should focus on replacing conventional strong acids and alkalis with biodegradable and recyclable soventes such as Deep Eutectic Solvents (DES), which can efficiently cleave disulfide bonds under moderate temperatures with a reduced environmental footprint.

## 5. Conclusions

This study conducted a bibliographic and bibliometric analysis of scientific articles regarding the utilization of pretreated chicken feather substrate for digestion. The search was conducted using the Scopus database and included original articles published until 2024.

The bibliometric analysis of the 24 retrieved articles highlighted a consistency over the years regarding scientific production on the digestion of chicken feather waste. It was also found that Sweden (5), China (3), and Nigeria (2) are the countries with the most published works in this field. In these articles, the most common keywords used by authors and journals were “chicken feather”, “methane”, “article”, “anaerobic digestion” and “animal” indicating a strong relationship between the method (anaerobic digestion), the raw material (chicken feather), and the product (methane).

The analysis identified that studies in this field can be broadly grouped into anaerobic digestion, co-digestion with food waste and/or animal manure, use of slaughterhouse waste, and biohydrogen production, with most of them based on chicken feather co-digestion.

Additionally, keratin, a primary protein found in chicken feathers, plays a significant role in these studies. Its complex and resilient structure necessitates effective pretreatment methods to enhance its biodegradability during anaerobic digestion. The conversion of keratin into simpler compounds is essential for efficient methane production. Furthermore, the insights gained from these studies could also contribute to advancements in polymer science. Polymers derived from keratin may present new avenues for sustainable material development, aligning with the growing interest in biopolymers and their applications across various industries.

Although feather pretreatment facilitates anaerobic conversion, transitioning to an industrial scale still faces significant technical challenges. Future research should prioritize: replacing corrosive chemical reagents with biological routes or green solvents such as Deep Eutectic Solvents (DES); optimizing anaerobic digestion parameters to mitigate the accumulation of free ammonia and volatile fatty acids (VFAs); and developing continuous pilot-scale bioreactors that closely simulate full-scale biogas plants.

## Figures and Tables

**Table 1:** The ten most productive countries based on the number of articles published in the Scopus database concerning the pretreatment of chicken feathers.

	Country	Citations	Number of Publications	Percentage (%)
1	Sweden	192	5	20.8
2	South Korea	103	1	4.2
3	Portugal	50	1	4.2
4	Nigeria	35	3	12.5
5	New Zealand	30	1	4.2
6	Brazil	29	1	4.2
7	United States	26	3	12.5
8	China	22	3	12.5
9	Argentina	20	1	4.2
10	Finland	18	1	4.2

**Table 2:** Top ten Institutions with the Highest Number of Publications on Pretreated Chicken Feathers in Biogas Production, Scopus Database.

University	Citations	Number of Publications	Country
University of Boras	174	4	Sweden
Pusan National University	103	1	South Korea
Chalmers University of Technology	100	2	Sweden
University of Minho	50	1	Portugal
Lanzatech Global	30	1	New Zealand
Scion	30	1	New Zealand
Federal University of Fronteira Sul	29	1	Brazil
Lagor State University	21	1	Nigerian
Universidade Nacional de La Plata	20	1	Argentina
Finniflag Oy	18	1	Finland

**Table 3:** The most used keywords of articles on Pretreated Chicken Feathers in Biogas Production, Scopus Database.

	Keyword	Frequency
1	Chicken Feather	17
2	Animal	13
3	Anaerobic digestion	11
4	Methane	10
5	Chicken	10
6	Metabolism	8
7	Biodegradation	8
8	Biogas	8
9	Keratinase	8
10	Hydrolysis	7

## Data Availability

The data presented in this study are available on request from the corresponding author. The raw datasets are not publicly available due to database subscription, copyright, and licensing restrictions of the indexing platforms.

## References

[1] OECD-FAO (2025). OECD-FAO Agricultural Outlook 2025–2034.

[2] North M.O., Bell D.D. (1990). Commercial Chicken Production Manual.

[3] USDA FAS-United States Department of Agriculture, Foreign Agricultural Service (2025). Livestock and Poultry: World Markets and Trade. 2026 Global Meat Trade: Beef and Pork Languish, Chicken Continues Rise.

[4] Ningthoujam D.S., Tamreihao K., Mukherjee S., Khunjamayum R., Devi L.J., Asem R.S. (2018). Keratinaceous Wastes and Their Valorization Valorization through through Keratinolytic Microorganisms. Keratin.

[5] Limeneh D.Y., Tesfaye T., Ayele M., Husien N.M., Ferede E., Haile A., Mengie W., Abuhay A., Gelebo G.G., Gibril M. (2022). A Comprehensive Review on Utilization of Slaughterhouse By-Product: Current Status and Prospect. Sustainability.

[6] Pourjavaheri F., Ostovar S., Jones O.A.H., Smooker P.M., Brklja R., Sherkat F., Blanch E.W., Gupta A., Shanks R.A. (2019). Extraction of Keratin from Waste Chicken Feathers Using Sodium Sul Fi de and L-Cysteine. Process Biochem..

[7] Mattiello S., Guzzini A., Giudice A.D., Santulli C., Antonini M., Lupidi G., Gunnella R. (2023). Physico-Chemical Characterization of Keratin from Wool and Chicken Feathers Extracted Using Refined Chemical Methods. Polymers.

[8] Moore G.R.P., Martelli S.M., Gandolf C.A., Pires A.T.N., Laurindo J.B. (2006). Queratina de Penas de Frango: Extração, Caracterização e Obtenção de Filmes. Ciênc. Tecnol. Aliment..

[9] Ossai I.C., Hamid F.S., Hassan A. (2022). Valorisation of Keratinous Wastes: A Sustainable Approach towards a Circular Economy. Waste Manag..

[10] Tuly J.A., Ma H., Zabed H.M., Janet Q., Abiso E., Chen G., Ekumah J. (2023). Potentiality Assessment of Microbial Action on Combined Agri-Food Industrial Wastes in Amino Acids Catabolism. J. Funct. Foods.

[11] Zhou Y., Zeng G., Zhang F., Tang Z., Luo J., Li K., Li X. (2022). Preparation of Functional Fiber Hybrid Enhanced High Strength and Multifunctional Protein Based Adhesive. Mater. Des..

[12] Abdulmunem A.R., Mohd P., Sopian K., Hoseinzadeh S., Al-jaber H.A., Astiaso D. (2022). Waste Chicken Feathers Integrated with Phase Change Materials as New Inner Insulation Envelope for Buildings. J. Energy Storage.

[13] Elhamdi M., Belhadjletaief C., Hmidet N., Ghorbel S. (2025). Proteases and Keratinases from *Bacillus zhangzhouensis* MH1: Practical Use in Detergent, Leather, and Waste Management Processes. Int. J. Biol. Macromol..

[14] Zuo X., Yi P., Chen Q., Wu M., Zhang L., Pan B., Xing B. (2022). Inter-Molecular Interactions of Phthalic Acid Esters and Multi-Stage Sorption Revealed by Experimental Investigations and Computation Simulations. Chem. Eng. J..

[15] Deublein D., Setinhauser A. (2010). Biogas from Waste and Renewable Resources.

[16] Walsh J.L., Ross C.C., Smith M.S., Harper S.R., Wilkins W.A. (1988). Biogas Utilization Handbook.

[17] Gong C., Liu X., Jiang X. (2014). Application of Bacteriophages Specific to Hydrogen Sulfide-Producing Bacteria in Raw Poultry by-Products. Poult. Sci..

[18] van Eck N.J., Waltman L. (2018). VOSviewer Manual-Version 1.6.8..

[19] Vanaki P., Zaboli F., Kaboosi H., Savadkoohi F. (2024). Isolation and Identification of Keratinolytic Probiotic Bucillus Licheniformis Bacteria from the Soil below Poultry Slaughterhouse Waste. Braz. J. Biol..

[20] Laila U., ul Huda M., Shakoor I., Nazir A., Shafiq M., e Bareen F., Shaukat K., Alam T.M. (2024). A Novel Method for the Enhancement of Sunflower Growth from Animal Bones and Chicken Feathers. Plants.

[21] Zhang J., Mo S., Li H., Yang R., Liu X., Xing X., Hu Y., Li L. (2022). Veterinary Sciences Rothia Nasimurium as a Cause of Disease: First Isolation from Farmed Chickens. Vet. Sci..

[22] Kavun Y., Eken M. (2024). Investigation of Thermal, Acoustic, Mechanical, and Radiation Shielding Performance of Waste and Natural Fibers. Appl. Radiat. Isot..

[23] Deng S., Liu R., Li C., Xu X., Zhou G. (2021). Meat Quality and Fl Avor Compounds of Soft-Boiled Chickens: Effect of Chinese Yellow-Feathered Chicken Breed and Slaughter Age. Poult. Sci..

[24] IEA Bioenergy (2024). Country Reports: Implementation of Bioenergy in Sweden–2024 Update.

[25] Yam T.K. (2026). The Emerging US-China Energy Rivalry: Fossil Power vs Renewable Energy.

[26] Forgács G., Lundin M., Taherzadeh M.J., Horváth I.S. (2013). Pretreatment of Chicken Feather Waste for Improved Biogas Production. Appl. Biochem. Biotechnol..

[27] Patinvoh R.J., Feuk-lagerstedt E., Lundin M., Horváth I.S. (2016). Biological Pretreatment of Chicken Feather and Biogas Production from Total Broth. Appl. Biochem. Biotechnol..

[28] Costa J.C., Barbosa S.G., Sousa D.Z. (2012). Effects of Pre-Treatment and Bioaugmentation Strategies on the Anaerobic Digestion of Chicken Feathers. Bioresour. Technol..

[29] Strong P.J., Gapes D.J. (2012). Thermal and Thermo-Chemical Pre-Treatment of Four Waste Residues and the Effect on Acetic Acid Production and Methane Synthesis. Waste Manag..

[30] Forgács G., Alinezhad S., Mirabdollah A., Feuk-Lagerstedt E., Horváth I.S. (2011). Biological Treatment of Chicken Feather Waste for Improved Biogas Production. J. Environ. Sci..

[31] Schommer V.A., Wenzel B.M., Daroit D.J. (2020). Anaerobic Co-Digestion of Swine Manure and Chicken Feathers: Effects of Manure Maturation and Microbial Pretreatment of Feathers on Methane Production. Renew. Energy.

[32] Patinvoh R.J., Lundin M., Taherzadeh M.J., Sárvári Horváth I. (2020). Dry Anaerobic Co-Digestion of Citrus Wastes with Keratin and Lignocellulosic Wastes: Batch and Continuous Processes. Waste Biomass Valorization.

[33] Cavello I., Bezus B., Cavalitto S. (2021). The Keratinolytic Bacteria Bacillus Cytotoxicus as a Source of Novel Proteases and Feather Protein Hydrolysates with Antioxidant Activities. J. Genet. Eng. Biotechnol..

[34] Schwede S., Thorin E., Lindmark J., Klintenberg P., Suhonen A., Laatikainen R., Hakalehto E., Schwede S., Thorin E., Lindmark J. (2017). Using Slaughterhouse Waste in a Biochemical-Based Biorefinery–Results from Pilot Scale Tests. Environ. Technol..

[35] Mézes L., Tamás J. (2015). Feather Waste Recycling for Biogas Production. Waste Biomass Valorization.

[36] Sumardiono S., Bakti Jos A.A.E.D., Mahendra I., Cahyono H. (2021). Biogas Production from Coffee Pulp and Chicken Feathers Using Liquid- and Solid-State Anaerobic Digestions. Energies.

[37] Manga M., Aragón-Briceño C., Boutikos P., Semiyaga S., Olabinjo O., Muoghalu C.C. (2023). Biochar and Its Potential Application for the Improvement of the Anaerobic Digestion Process: A Critical Review. Energies.

[38] Costa J.M., Aguiar A.B.S., Montanari A.F.P., Damasceno B.G., Duran K.A., Jerônimo K.A., Silva M.M., Silva T.C.T., Rodriguez R.P. (2024). Environmental Aspects and Perspectives of the Brazilian Market for Biogas and Biomethane from Anaerobic Digestion: A Review. Bioenergy Res..

[39] El Salamony D.H., Hassouna M.S.E., Zaghloul T.I., He Z., Abdallah H.M. (2024). Bioenergy Production from Chicken Feather Waste by Anaerobic Digestion and Bioelectrochemical Systems. Microb. Cell Factories.

[40] Saba M., Khan A., Ali H., Bibi A., Gul Z., Khan A., Maqsood M., Rehman U., Badshah M., Hasan F. (2022). Microbial Pretreatment of Chicken Feather and Its Co-Digestion with Rice Husk and Green Grocery Waste for Enhanced Biogas Production. Front. Microbiol..

[41] Kannan P.S., Muthukannan M., Ganesh K., Janani R. (2024). Effect of Anaerobic Co-Digestion of Waste Chicken Feather with Banana Stem Juice for Production of Biogas. Biomass Convers. Biorefinery.

[42] Sun L., Tang D., Tai X., Wang J., Long M., Xian T., Jia H., Wu R., Ma Y., Jiang Y. (2023). Effect of Composted Pig Manure, Biochar, and Their Combination on Antibiotic Resistome Dissipation in Swine Wastewater-Treated Soil. Environ. Pollut..

[43] Tamreihao K., Mukherjee S., Khunjamayum R., Devi L.J., Asem R.S., Ningthoujam D.S. (2019). Feather Degradation by Keratinolytic Bacteria and Biofertilizing Potential for Sustainable Agricultural Production. J. Basic Microbiol..

[44] Yusuf I., Arzai K.I., Dayyab A.S. (2020). Evaluation of Pre-Treatment Methods and Anaerobic Co-Digestions of Recalcitrant Melanised Chicken Feather Wastes with Other Wastes for Improved Methane and Electrical Energy Production. Jordan J. Biol. Sci..

[45] Li K., Yang Y., Zhang S., Cheng X. (2022). Dynamics of the Bacterial Community’s Soil During the In-Situ Degradation Process of Waste Chicken Feathers. Indian J. Microbiol..

[46] Revankar A.G., Bagewadi Z.K., Bochageri N.P., Yunus Khan T.M., Mohamed Shamsudeen S. (2023). Response Surface Methodology Based Optimization of Keratinase from *Bacillus velezensis* Strain ZBE1 and Nanoparticle Synthesis, Biological and Molecular Characterization. Saudi J. Biol. Sci..

[47] Miserez A., Soon W.L., Peydayesh M., De Wild T., Donat F., Saran R., Müller C., Gubler L., Mezzenga R. (2023). Renewable Energy from Livestock Waste Valorization:Amyloid-Based Feather Keratin Fuel Cells. ACS Appl. Mater. Interfaces.

[48] Kriswantoro J.A., Tseng C.H., Fois F., Chu C.Y., Manzo E., Petracchini F. (2025). Synergetic Hydrogen and Methane Productions from Anaerobic Digestion of Selected Rural-Farming Plant and Animal-Based Biomass Wastes. Korean J. Chem. Eng..

[49] Lema J.M., Méndez R. (1997). Tratamientos Biológicos Anaerobios. Contaminación e Ingeniéria Ambiental.

[50] Dinesh G.H., Murugan R.S., Arumugam N., Basu M.J. (2019). Anaerobic Process for Biohydrogen Production Using Keratin Degraded Effluent. J. Pure Appl. Microbiol..

[51] Suharti S., Rozaq H.N., Qisti A., Alvionita M., Wonorahardjo S. (2023). Keratinase Production by *Bacillus* sp. MD24 in Sub-Merge and Solid State Fermentation. Malays. J. Fundam. Appl. Sci..

[52] Iwuozor K.O., Abdulkareem S.A., Amoloye M.A., Emenike E.C., Ezzat A.O., Mustapha J.A., Egbemhenghe A.U., Adeniyi A.G. (2024). Eco-Friendly Composite Materials: Enhancing Sustainability with Sugarcane Bagasse Biochar and Polystyrene Resin. Sugar Tech.

